# Association between hypodontia of permanent maxillary lateral incisors and other dental anomalies

**DOI:** 10.1590/2177-6709.25.6.069-078.bbo

**Published:** 2020

**Authors:** Diego Junior da Silva Santos, José Augusto Mendes Miguel

**Affiliations:** 1 Private practice (Rio de Janeiro/RJ - Brazil).; 2 Universidade do Estado do Rio de Janeiro, Undergraduate Dentistry Course and Master's Program in Dentistry (Rio de Janeiro/RJ, Brazil).

**Keywords:** Hypodontia, Anodontia, Tooth agenesis, Tooth abnormalities

## Abstract

**Introduction:**

Tooth agenesis is often associated with other tooth anomalies, such as microdontia, delayed eruption and ectopic eruption. Moreover, they may be found all in the same individual, as certain genetic mutations may have a variable phenotypic expression. Treatment of cases of hypodontia of anterior teeth should not involve only opening or closing space for prosthetic rehabilitation. Individuals with hypodontia of permanent maxillary lateral incisors may have teeth with a mesiodistal width smaller than that of patients with a normal dentition, and which may need reshaping to achieve an esthetic and functional occlusion.

**Objective:**

This clinical case report discusses the association of hypodontia of permanent maxillary lateral incisors with other tooth anomalies and their treatment alternatives.

## RESUMO

**Introdução:**

As agenesias dentárias são frequentemente associadas a outras anomalias dentárias, tais como microdontia, atraso na erupção e ectopismo. Além disso, podem vir todas juntas em um mesmo indivíduo, pois certas mutações genéticas podem se expressar fenotipicamente de diferentes formas. A abordagem terapêutica nos casos de hipodontia de dentes anteriores não deve se pautar somente entre fechar ou abrir espaço para substituição protética. Portadores de hipodontia de incisivos laterais superiores permanentes podem apresentar os outros dentes com largura mesiodistal menor que os de pacientes com dentição normal, e podem necessitar de reanatomizações para que se obtenha uma oclusão dentária estética e funcional.

**Objetivo:**

Dessa forma, o objetivo do presente artigo é relatar um caso clínico e discutir a associação da hipodontia de incisivos laterais superiores permanentes com outras anomalias dentárias, e suas alternativas de tratamento.

## INTRODUCTION

Tooth agenesis, one of the most common tooth anomalies, is the absence of teeth due to a failure in their development.^1^

Several factors may affect the normal development of tissues and lead to changes and defects in tooth shape and size. The causes of tooth anomalies may be congenital, developmental or acquired.^2^

Tooth agenesis is classified according to the number of missing teeth. Hypodontia is the term used to describe the absence of one to five teeth; oligodontia, the absence of six or more teeth; and anodontia, the absence of all teeth.^3^

These terms may be confusing to clinical dentists when talking to each other or to their patients. Several times tooth agenesis is used to indicate congenitally missing teeth. However, the term congenitally missing is inadequate to describe this clinical entity, as tooth development is completed after birth.^4^ Hypodontia is etymologically more adequate to classify agenesis when only one tooth is missing, whereas oligodontia and anodontia are more appropriate to describe severe forms of tooth agenesis.^5^

Tooth agenesis is rare in primary dentition. Hypodontia of a primary tooth is associated with hypodontia of its permanent successor. The presence of a primary tooth does not necessarily mean that its permanent successor will also be present. However, hypodontia of a primary tooth is followed by hypodontia of its permanent successor. This association is explained by the histology of odontogenesis: the permanent tooth develops from the tooth bud attached to the dental papilla of the primary tooth under formation. Therefore, the absence of the dental papilla of the primary tooth means that the tooth bud of the permanent successor is missing too.

Tooth agenesis is an anomaly that may be associated with several syndromes, such as Down syndrome, ectodermal dysplasia, Axenfeld-Rieger syndrome, radiotherapy and hypophosphatasia.^2^ Genetic inheritance is the main etiologic factor of tooth agenesis. However, this entity has a multifactorial character associated with genetic characteristics, endocrine dysfunctions, viral problems, trauma and congenital deformities, which are mentioned as the main causes of agenesis in the literature.

The prevalence of agenesis of permanent teeth in non-syndromic individuals is higher among Europeans (4.6% men; 5.5% women) and Australians (5.5 men; 7.6% women) and lower among American white people (3.2% men; (4.6% women).^6^ The prevalence of agenesis of permanent maxillary lateral incisors ranges from 6% to 8% in different ethnic groups, and molecular genetics has identified shared genetic mutations in families with tooth agenesis.^7^ Moreover, individuals with agenesis of permanent maxillary lateral incisors or other teeth often also have other tooth anomalies. That is, the same genetic mutation may have a variable phenotypical expression.^8^

Permanent maxillary lateral incisors are the teeth with the second most frequently affected with hypodontia. Treatment alternatives for this type of tooth anomaly are: space closure by mesialization of permanent canine, placement of a resin-bonded prosthesis, placement of osseointegrated implants, or autogenous tooth transplantation.^7,9-11^

The absence of permanent teeth may, for example, generate problems in the articulation of dental arches, a predisposing factor to malocclusion, and may lead to important changes in the stomatological system. Moreover, it is associated with great esthetic discomfort, which is the main complaint of patients with agenesis of maxillary lateral incisors. 

However, treatment approaches in cases of hypodontia of permanent maxillary lateral incisors should not be based only on whether to open or close space for prosthetic rehabilitation. An accurate diagnosis and multidisciplinary planning should define the best treatment option. The mesiodistal width of the other teeth is smaller in patients with hypodontia of maxillary lateral incisors.^12^ The other teeth often require reshaping so that their mesiodistal diameter is appropriate for an esthetic and functional dental occlusion. Moreover, an ideal occlusion is dependent on the presence of a well-proportioned anatomy of both maxillary and mandibular teeth, so that dental alignment is ideal when associated with the closure of the space resulting from hypodontia.^13^

This study discusses the association of hypodontia of permanent maxillary lateral incisors with other tooth anomalies, and describes treatment alternatives to treat the absence of maxillary lateral incisors. A brief review of the literature is followed by the description of the clinical case of a patient with hypodontia and microdontia of permanent maxillary lateral incisors.

## CASE REPORT

**Figure 1:**
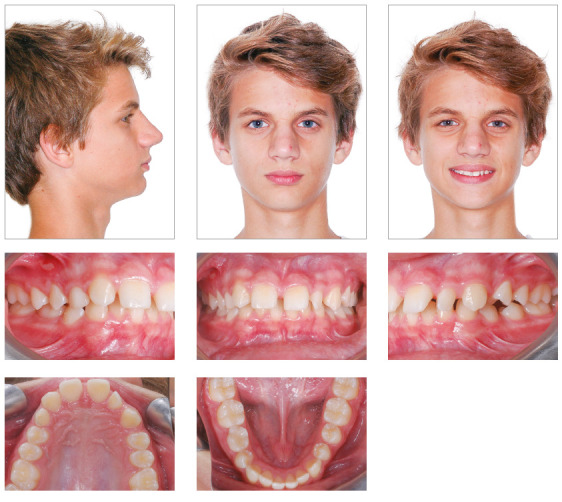
Pretreatment photographs.

**Figure 2:**
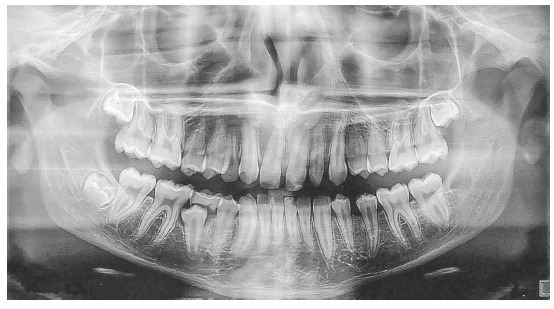
Pretreatment panoramic radiograph.

**Figure 3:**
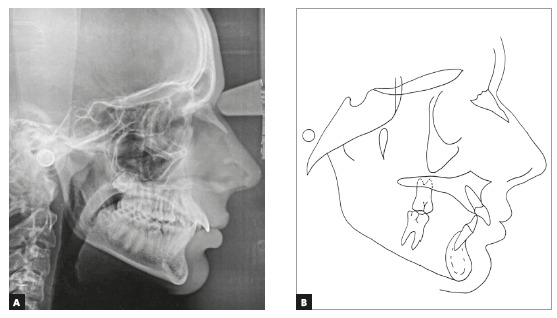
Pretreatment cephalometric radiograph (A) and cephalometric tracing (B).

A white 14-year and 2-month-old boy presented with a chief complaint of diastema of anterior maxillary teeth, missing tooth #12, microdontia of tooth #22 and delayed eruption of tooth #53. Clinical examination revealed that tooth #13 had erupted at the site of the missing tooth #12, and that the eruption of tooth #53 was delayed and distal to tooth #13. The patient had microdontia of tooth #22, and tooth #85 had not erupted yet (Figs 1, 2). He had a Class I skeletal pattern (ANB = 4^o^), a mesocephalic pattern, a balanced vertical growth pattern (SN.GoGn = 32^o^, y-axis = 52^o^, FMA = 21^o^), a well-positioned maxilla and a retrognathic mandible (SNA = 81^o^ and SNB = 77^o^). He also had an edge-to-edge molar relationship, which tended to an Angle Class II occlusion, and positive space discrepancy in the maxillary arch (6.5 mm). Overbite and overjet were increased (4.67 mm and 8 mm), maxillary dental midline was deviated 3.5 mm to the right, and there were diastemas between maxillary anterior teeth. Maxillary incisors were proclined (1.NA = 35^o^, 1-NA = 5 mm) and mandibular incisors were slightly crowded and proclined (1.NB = 27^o^, 1-NB = 6mm) (Table 1, Fig 3). He had a straight profile and an upper lip short to the S-line (Steiner), a right mentolabial angle and an obtuse nasolabial angle. The analysis of function revealed atypical phonation and deglutition, as well as tongue thrust. 

**Table 1:** Cephalometric values before (A) and after (B) treatment.

	Measurement		Normal	A	B	Dif. A/B
Skeletal pattern	SNA	(Steiner)	82°	81°	80°	1
	SNB	(Steiner)	80°	77°	79°	2
	ANB	(Steiner)	2°	4°	1°	3
	Wits	(Jacobson)	♀ 0 ± 2mm	5mm	0mm	5
			♂ 1 ± 2mm			
	Angle of Convexity	(Downs)	0°	4°	-6°	10
	Eixo Y	(Downs)	59°	52°	54°	2
	Facial Angle	(Downs)	87°	80°	90°	10
	SN.GoGn	(Steiner)	32°	32°	27°	5
	FMA	(Tweed)	25°	21°	18.5°	2.5
Dental pattern	IMPA	(Tweed)	90°	96.5°	96°	0.5
	1.NA (degrees)	(Steiner)	22°	35°	28°	7
	1-NA (mm)	(Steiner)	4 mm	5mm	4mm	1
	1.NB (degrees)	(Steiner)	25°	27°	23°	4°
	1-NB (mm)	(Steiner)	4 mm	6mm	4mm	2
	- Interincisal Angle	(Downs)	130°	114°	128°	14
	1 - APg	(Ricketts)	1 mm	0mm	0mm	0
Profile	Upper lip-S line	(Steiner)	0	-1mm	-6mm	5
	Lower lip-S line	(Steiner)	0	0mm	-5mm	5

Treatment objectives were leveling and alignment of dental arches, adequate overbite and overjet, coinciding dental and facial midlines, molar and canine Class I relationship, reshaping of tooth #22 and space opening for implant at the site of the missing tooth #12.

## TREATMENT AND ORTHODONTIC MECHANICS

Multidisciplinary planning included opening space for the replacement of missing tooth #12 and reshaping of #22. The patient’s guardians were informed that this treatment strategy would require a longer time, because the use of asymmetric mechanics would be necessary. Moreover, the treatment would require total patient compliance with the use of intermaxillary elastics to achieve an adequate dental occlusion. They were also informed that an osseointegrated implant would have to be placed when the patient reached adulthood, and that the long-term stability of soft and hard tissues around the implant, as well as esthetic results, were unpredictable.^9^

**Figure 4:**
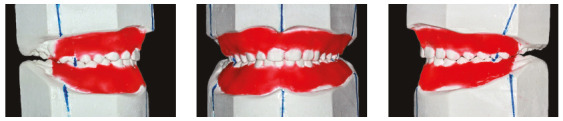
Orthodontic setup using plaster models.

The final width of tooth #22, affected by microdontia, was calculated using the simplified method described by German et al,^13^ which defines that the mesiodistal widths of anterior teeth are correlated to each other and may be easily estimated according to the mesiodistal diameter of mandibular incisors. Tooth size was then used to build the orthodontic setup with plaster models to plan treatment mechanics and to check the viability of achieving the final estimated mesiodistal width. Tooth #22, affected by microdontia, had a mesiodistal width of 5.45 mm. The orthodontic setup included a 1-mm increase for tooth #22. This way a molar and canine Class I occlusion and adequate overbite and overjet might be achieved (Fig 4). 

**Figure 5:**
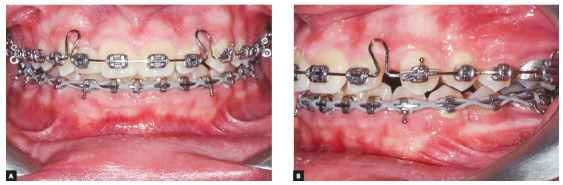
Frontal intraoral photo (A) and left lateral intraoral photo (B) during maxillary incisor retraction.

After the placement of the fixed appliance, a sequence of preformed nickel-titanium archwires were used for leveling and alignment. The space at the site of missing tooth #12 was obtained using open NiTi coil springs. Class II intermaxillary elastics in the right side and Class III in the left side were used after the correction of maxillary dental midline deviation. After the space was opened, a prefabricated provisional was attached to a 0.019 x 0.025-in rectangular stainless steel archwire with Bull retraction loops. Retraction to close diastemas and maintain the space distal to tooth #22 was controlled, so that the tooth could be reshaped later (Fig 5). 

For retention after the fixed appliance was removed, a lingual canine-to-canine 0.7-mm stainless steel arch was bonded to the mandibular teeth, and a removable wraparound retainer was used for the maxillary teeth. The patient received instructions to use the removable retainer full time for six months and only overnight after that.^14^ A provisional for the missing tooth #12 was placed in the mouth using a Maryland bridge and removed only when the osseointegrated implant was placed.

## RESULTS

**Figure 6:**
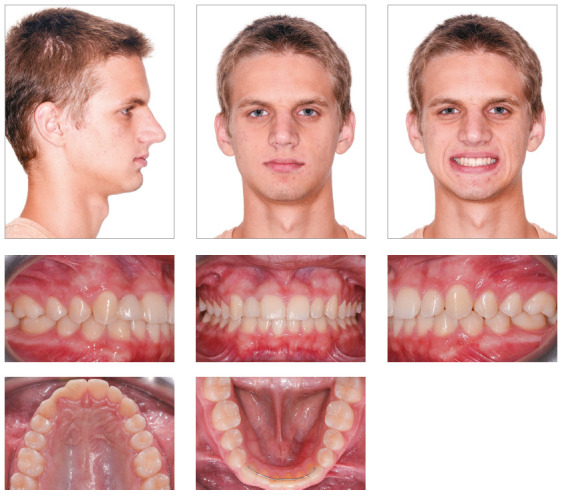
Posttreatment photographs.

All the treatment objectives were achieved: molar and canine Class I relationship; maintenance of lip position and profile; adequate overbite and overjet; midline correction; space for the implant; and an adequate mesiodistal diameter for tooth #22 (Fig 6).

**Figure 7:**
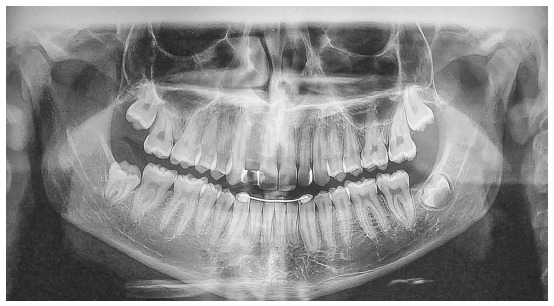
Posttreatment panoramic radiograph.

**Figure 8:**
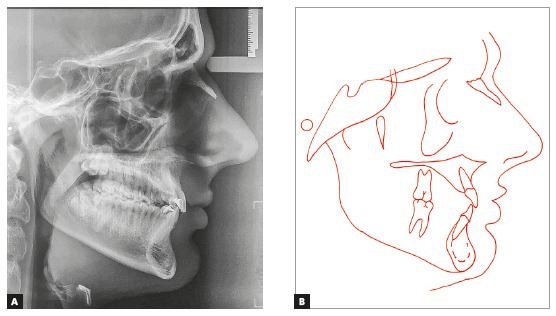
Posttreatment cephalometric radiograph (A) and cephalometric tracing (B).

**Figure 9:**
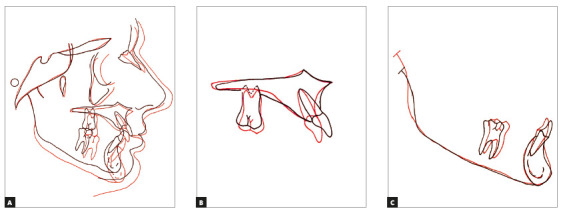
Total (A) and partial (B, C) superimpositions of cephalometric tracings pretreatment (black) and posttreatment (red).

The profile became more concave, but its satisfactory esthetic appearance was preserved. Roots were parallel at the end of the treatment, and the anteroposterior relationship between maxilla and mandible improved (ANB = 1^o^). Mandibular growth was satisfactory, and the balanced pattern of facial growth was preserved (SN.GoGn = 27^o^, y-axis = 54^o^, FMA = 18.5^o^) (Table 1, Figs 7 and 8). Superimpositions showed that the patient maintained his balanced facial and mandibular growth, and that the maxillary incisors were retroclined as a result of the orthodontic mechanics used for retraction (Fig 9). 

**Figure 10:**
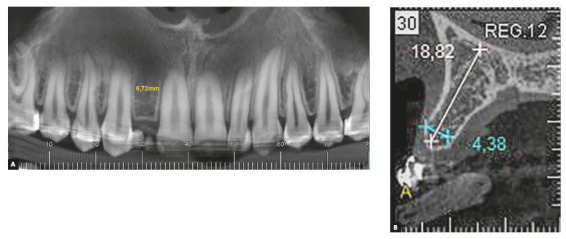
Cone-beam CT slices. Panoramic view shows interradicular distance between teeth #13 and #11 (A). Sagittal view shows anteroposterior and vertical bone thicknesses in region of missing tooth #12 (B).

Interradicular distance at the apices of teeth #11 and #13 for the placement of an implant in the space of missing tooth #12 was 6.72 mm. Olsen and Kokich^15^ found that the adequate interradicular distance between maxillary canine and central incisor to place an implant is at least 5.7 mm, whereas intercoronal space should be 6.3 mm (Fig 10). 

## DISCUSSION

Studies in molecular genetics have found mutations in the MSX1, PAX9 and AXlN2 genes in families with multiple cases of tooth agenesis.^15,16^ The transmission or familial inheritance of these genetic mutations may be the result of dominant, recessive or X-linked recessive disorders. Some homeobox genes, such as MSX1, MSX2, PAZ 9 and TGFA, have an important role in the development of dentition and in craniofacial morphogenesis.^7^ Moreover, hypodontia of permanent maxillary lateral incisors or other teeth are often associated with other tooth anomalies in the same patient: microdontia, delayed eruption, ectopic eruption, and others. This indicates that different tooth anomalies in the same individual at any one time may be distinct expressions of the same genetic mutation.^16^ In the clinical case described here, the patient had an anomaly of number and shape: hypodontia of tooth #12 and microdontia of tooth #22.

In this clinical case, tooth #23 erupted normally, but tooth #13 erupted in the site of missing tooth #12. Canines are the teeth that have the longest path of eruption: about 22 mm until they reach their final occlusal position. This distance is a risk factor for deviations during their eruption.^16^ Canine eruption may be explained by the *Guidance Theory*, which indicates that permanent lateral incisors are eruption guides for canines. If incisors have any shape anomaly or are missing, canines may erupt ectopically or become impacted.^18^

Orthodontic patients with hypodontia of lateral incisors are a challenge for a satisfactory orthodontic treatment completion, because ideal intercuspation at the end of the treatment is dependent on the relationship of crown sizes between maxillary and mandibular teeth. In such cases, satisfactory final intercuspation is difficult, as hypodontia of lateral incisors is often associated with a reduced mesiodistal width of other teeth.^19^ Consequently, drilling or interproximal augmentation of the crowns of adjacent teeth may be necessary in individuals with hypodontia of maxillary lateral incisors.^20^

In this case report, tooth proportions were corrected in the anterior segment, where the main problem was. Several methods may be used as a reference for orthodontists when defining the ideal size of a tooth crown. A study conducted by Black^21^ was one of the first to measure teeth, and the tables of tooth sizes in that study are still used today. 

The golden proportion has also been suggested for the calculation of ideal tooth size. However, the application of the golden proportion for dental rehabilitations has been refuted in several studies that found patients and dentists were unhappy with the smiles achieved when using this technique. Such dissatisfaction is a result mainly of cases of narrow lateral incisors, in which teeth that measure 3 to 4 mm less than ideal are classified as less appealing by laypeople and specialists.^21^ ^,^ ^22^

In the case report presented here, a simplified version of the method described by German et al.^13^ was used to adapt the mesiodistal width of tooth #22 to the width of the prosthesis for missing tooth #12. This protocol ensures a simplified communication with the prosthesis technician by means of calculations based on the correlation between the mesiodistal widths of the crowns of anterior teeth. The results of these calculations should not be analyzed as absolute values, but, rather, as a reference for the orthodontist during planning with setups using either plaster or virtual models.^13^ The calculation of the proportions of the ideal size of teeth should take into consideration also the vertical dimension of the crowns. Câmara^23^ reported that it is common to use the following measurements as a reference for the crowns of maxillary permanent central incisors: width = 8 to 9 mm; height = 10 to 11 mm. These values may be used as initial parameters for predictions. However, more important than the use of isolated measurements are the mean proportions between coronal height and width, which may range from 70% to 80%.^24^

The growth of the nose and in the region of the pogonion, added to the counterclockwise rotation of the mandible, were the main contributions to the increase of the patient’s profile concavity. The growth of the nose bone is completed at about 10 years of age. After that, nose growth is limited to the nasal cartilage and soft tissues, which undergo accelerated growth in adolescence. In this phase, the nose becomes more prominent, especially in boys (Fig 9).^25^

The idea that incisor retraction alone flattens facial profile is not a consensus in the literature, except in cases of extraction of the four premolars when incisors are retracted.^26^ Even when planning does not include extractions, the reduction of overjet by incisor retraction may result in lip retrusion. Lip position is closely associated with the degree of mandibular incisor inclination: when mandibular incisors are proclined, they may limit the degree of overjet and, consequently, the degree of incisor and lip retraction. Therefore, in this clinical case, the side effect of mandibular incisor proclination due to Class II intermaxillary elastic mechanics contributed to a reduction of overjet and, consequently, to a lower degree of incisor and lip retraction and the preservation of facial profile harmony.

The clinical association between mandibular incisor proclination and gingival recession has not been definitely explained, and few studies have reported on long-term effects of mandibular incisor proclination on the periodontium.^27^ A marked proclination of mandibular incisors may be achieved without the risk of gingival recession.^28^ The gingival characteristics of the anteroinferior segment of the patient in this clinical study ensured the safety of mandibular incisor proclination, resulting in about 2 mm of the attached gingiva and good plaque control.^29^ Whether to close or open space for the replacement of a missing tooth has always been a dilemma for a clinical dentist, but, according to Zachrisson et al.^9^, space closure for lateral incisors by means of canine migration leads to better long-term results. This author also added that it is not possible to predict the degree of complications that affect hard and soft tissues around the osseointegrated implant-supported crowns, which may compromise esthetics mainly.^9^

 However, in the case described here, the alternative of rehabilitation using an adhesive prosthesis would require drilling adjacent teeth to receive the prosthesis. Space closure by means of mesial migration of posterior teeth toward tooth #13 would result in a longer treatment time and higher costs because of the need to use temporary anchorage or to apply techniques that require total patient cooperation.

The ideal age for the surgery for implant placement for missing tooth #12 should be assessed frequently by the dentist to define whether bone maturity is satisfactory for the procedure. In most cases, girls at the age of 16 years and boys at 21 may already undergo surgery for implant placement.^30^

## CONCLUSIONS

» Hypodontia of permanent maxillary lateral incisors may be associated with other forms of tooth anomalies. This entity is transmitted by familial heritance in a dominant, recessive or X-linked recessive manner. 

» Two treatment options are recommended for cases of agenesis of maxillary lateral incisors: space closure and mesial movement of canines to the position of the missing incisors, or the opening or preservation of spaces for prosthetic rehabilitation of the missing lateral incisors using implants. A multidisciplinary approach should be adopted to correct mesiodistal widths of anterior teeth, to achieve a satisfactory esthetic and functional outcome.
